# Neurocognitive trajectory and proteomic signature of inherited risk for Alzheimer’s disease

**DOI:** 10.1371/journal.pgen.1010294

**Published:** 2022-09-01

**Authors:** Manish D. Paranjpe, Mark Chaffin, Sohail Zahid, Scott Ritchie, Jerome I. Rotter, Stephen S. Rich, Robert Gerszten, Xiuqing Guo, Susan Heckbert, Russ Tracy, John Danesh, Eric S. Lander, Michael Inouye, Sekar Kathiresan, Adam S. Butterworth, Amit V. Khera

**Affiliations:** 1 Program in Medical and Population Genetics, Broad Institute of MIT and Harvard, Cambridge, Massachusetts, United States of America; 2 Cambridge Baker Systems Genomics Initiative, Department of Public Health and Primary Care, University of Cambridge, Cambridge, United Kingdom; 3 Cambridge Baker Systems Genomics Initiative, Baker Heart & Diabetes Institute, Melbourne, Victoria, Australia; 4 British Heart Foundation Cardiovascular Epidemiology Unit, Department of Public Health and Primary Care, University of Cambridge, Cambridge, United Kingdom; 5 British Heart Foundation Centre of Research Excellence, University of Cambridge, Cambridge, United Kingdom; 6 National Institute for Health Research Cambridge Biomedical Research Centre, University of Cambridge and Cambridge University Hospitals, Cambridge, United Kingdom; 7 The Institute for Translational Genomics and Population Sciences, Department of Pediatrics, The Lundquist Institute for Biomedical Innovation at Harbor-University of California, Los Angeles Medical Center, Torrance, California, United States of America; 8 Center for Public Health Genomics, University of Virginia, Charlottesville, Virginia, United States of America; 9 Division of Cardiovascular Medicine, Beth Israel Deaconess Medical Center and Harvard Medical School; 10 Department of Epidemiology, University of Washington, Seattle, Washington, United States of America; 11 Department of Biochemistry, Larner College of Medicine, University of Vermont, Burlington, Vermont, United States of America; 12 Health Data Research UK Cambridge, Wellcome Genome Campus and University of Cambridge, Cambridge, United Kingdom; 13 Department of Human Genetics, Wellcome Sanger Institute, Hinxton, United Kingdom; 14 National Institute for Health Research Blood and Transplant Research Unit in Donor Health and Genomics, University of Cambridge, Cambridge, United Kingdom; 15 Department of Biology, Massachusetts Institute of Technology, Cambridge, Massachusetts, United States of America; 16 Department of Systems Biology, Harvard Medical School, Boston, Massachusetts, United States of America; 17 Department of Clinical Pathology, University of Melbourne, Parkville, Victoria, Australia; 18 The Alan Turing Institute, London, United Kingdom; 19 Verve Therapeutics, Cambridge, Massachusetts, United States of America; 20 Division of Cardiology and Center for Genomic Medicine, Department of Medicine, Massachusetts General Hospital, Boston, Massachusetts, United States of America; 21 Department of Medicine, Harvard Medical School, Boston, Massachusetts, United States of America; Stanford University, UNITED STATES

## Abstract

For Alzheimer’s disease–a leading cause of dementia and global morbidity–improved identification of presymptomatic high-risk individuals and identification of new circulating biomarkers are key public health needs. Here, we tested the hypothesis that a polygenic predictor of risk for Alzheimer’s disease would identify a subset of the population with increased risk of clinically diagnosed dementia, subclinical neurocognitive dysfunction, and a differing circulating proteomic profile. Using summary association statistics from a recent genome-wide association study, we first developed a polygenic predictor of Alzheimer’s disease comprised of 7.1 million common DNA variants. We noted a 7.3-fold (95% CI 4.8 to 11.0; p < 0.001) gradient in risk across deciles of the score among 288,289 middle-aged participants of the UK Biobank study. In cross-sectional analyses stratified by age, minimal differences in risk of Alzheimer’s disease and performance on a digit recall test were present according to polygenic score decile at age 50 years, but significant gradients emerged by age 65. Similarly, among 30,541 participants of the Mass General Brigham Biobank, we again noted no significant differences in Alzheimer’s disease diagnosis at younger ages across deciles of the score, but for those over 65 years we noted an odds ratio of 2.0 (95% CI 1.3 to 3.2; p = 0.002) in the top versus bottom decile of the polygenic score. To understand the proteomic signature of inherited risk, we performed aptamer-based profiling in 636 blood donors (mean age 43 years) with very high or low polygenic scores. In addition to the well-known apolipoprotein E biomarker, this analysis identified 27 additional proteins, several of which have known roles related to disease pathogenesis. Differences in protein concentrations were consistent even among the youngest subset of blood donors (mean age 33 years). Of these 28 proteins, 7 of the 8 proteins with concentrations available were similarly associated with the polygenic score in participants of the Multi-Ethnic Study of Atherosclerosis. These data highlight the potential for a DNA-based score to identify high-risk individuals during the prolonged presymptomatic phase of Alzheimer’s disease and to enable biomarker discovery based on profiling of young individuals in the extremes of the score distribution.

## Author summary

Alzheimer’s disease is a leading cause of dementia and global morbidity. Despite decades of research, disease modifying therapies remain elusive. One possible explanation for failed clinical trials is intervention too late in the disease process when therapies are unlikely to be effective. Here, we developed a genetic predictor for Alzheimer’s disease allowing us to identify asymptomatic individuals at increased risk of developing Alzheimer’s disease. We next measured the levels of 3,231 proteins in the blood of middle-aged, healthy individuals and found proteins whose levels were changed in individuals with a high genetic risk of developing Alzheimer’s disease. Several of these proteins have not previously been studied in Alzheimer’s. Our study suggests a method to identify high genetic risk individuals during the presymptomatic phase of disease, enabling us to discover new protein-based biomarkers in the early stages of disease progression.

## Introduction

Alzheimer’s disease is a neurodegenerative disorder characterized by slowly progressive impairment in memory and executive function, with a lifetime risk of up to 10% [1]. Although clinical diagnosis typically occurs late in life, the pathologic hallmarks–including neuritic plaques and neurofibrillary tangles–begin to accumulate during a prolonged presymptomatic phase [2,3]. Risk stratification using advanced neuroimaging [4–7] or biomarker assessment from cerebrospinal fluid is possible [8–12], but is resource-intensive or invasive, and is unlikely to be useful when applied to asymptomatic individuals early in life [13]. Although some treatments can improve symptoms, no disease-modifying therapies are currently available [14,15].

For a range of conditions, patient stratification based on inherited DNA variation has proven useful in providing insights into disease biology or enabling targeted therapy [16]. The traditional approach has relied on rare, ‘monogenic’ variants of large effect that disrupt a specific physiologic pathway. For Alzheimer’s disease, causative variants in three key genes–amyloid precursor protein (*APP*) [17–19], presenilin 1 (*PSEN1*) [20], and presenilin 2 (*PSEN2*) [21]–were uncovered in studies of families enriched for early-onset cases. These observations have provided key insight into the role of amyloid precursor protein secretion and cleavage abnormalities that accelerate disease but are present in fewer than 5% of afflicted individuals [22].

A second approach to DNA-based risk stratification involves polygenic scoring, which integrates information from many variants that confer individually modest increases in risk via many different pathways. Advances in polygenic score development have demonstrated potential clinical utility for several important and preventable diseases, identifying–in some cases–individuals with risk equivalent to rare monogenic mutations [23–25].

**Fig 1:**
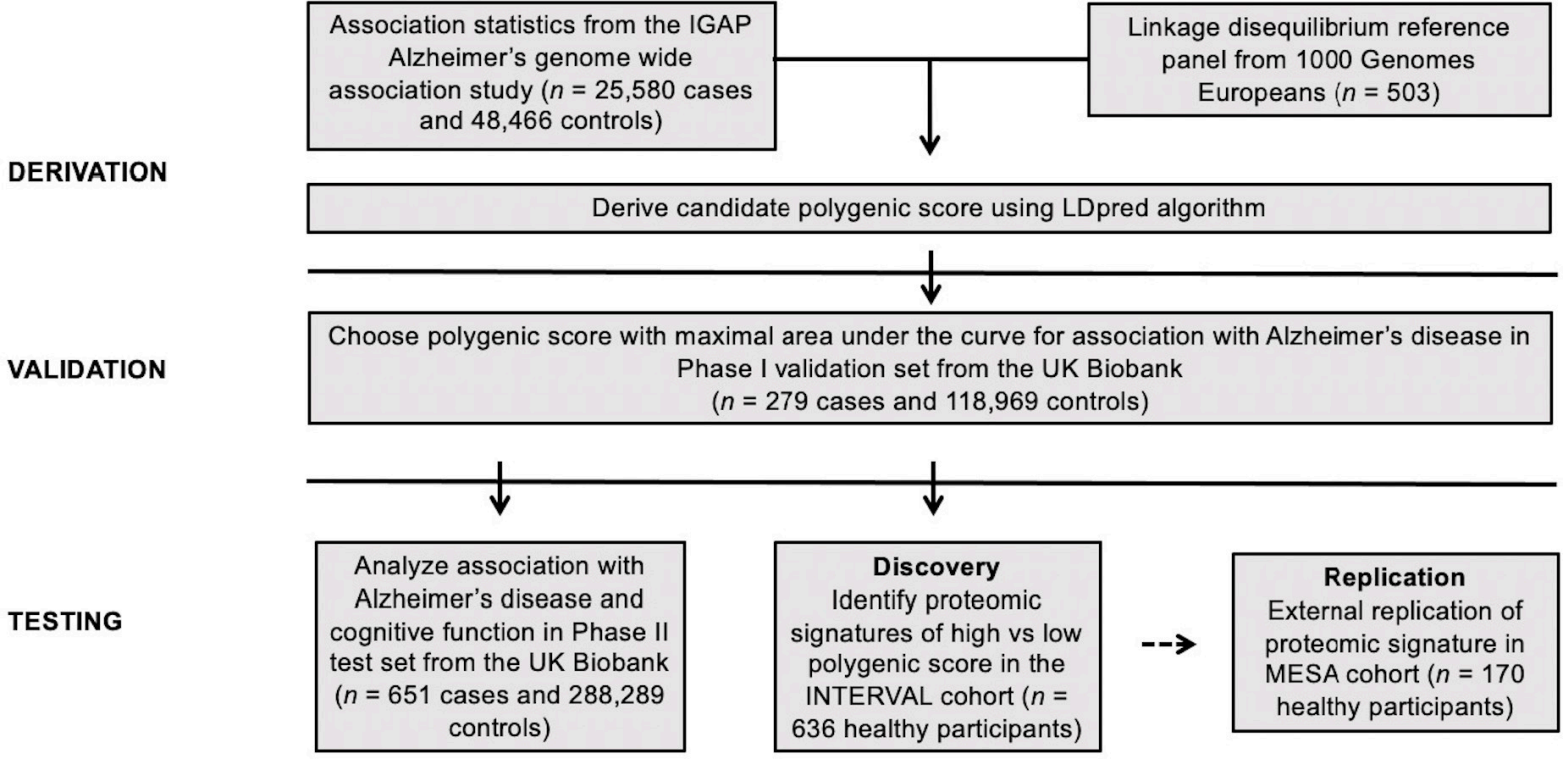
Study Design and Workflow. Using previously published genome-wide association study summary association statistics [26] and a linkage disequilibrium reference panel of 503 European-ancestry participants from the 1000 Genomes study [27], we derived six candidate polygenic scores for Alzheimer’s disease using the LDPred computational algorithm [28]. The best performing polygenic score was selected based on maximal area-under-the curve in a validation dataset derived from the UK Biobank [29] (n = 119,248 European-ancestry participants) and subsequently calculated in an independent set of UK Biobank participants (n = 288,940). Associations with a clinical diagnosis of Alzheimer’s and performance on a neurocognitive test were determined in both overall and in age-stratified analyses. In an independent dataset derived from the INTERVAL study of healthy blood donors [30], we compared the levels of 3,231 circulating proteins between 636 participants in the top or bottom decile of the polygenic score. We sought to replicate proteins significantly associated with the polygenic score in the INTERVAL study in participants of the MESA study. IGAP: International Genomics of Alzheimer’s Project [26]; UKBB: United Kingdom Biobank [29]; MESA: Multi-Ethnic Study of Atherosclerosis [31].

Here, we set out to derive and validate a new polygenic score for Alzheimer’s disease to test two key hypotheses: (i) a polygenic score can stratify the population into differing trajectories of clinical and subclinical cognitive decline with age; (ii) proteomic profiling of asymptomatic individuals with high or low polygenic score may nominate new circulating biomarkers of disease (**Fig 1**).

## Results

To create a polygenic score, we used summary association statistics from a previously published genome-wide association (GWAS) study involving 21,982 AD cases and 41,944 unaffected controls and analyzing 7,055,881 common DNA variants [26]. Importantly, individuals in the UK Biobank study were not included in this previous GWAS. Summary statistics from more recent studies were not used because–although they were larger–they included participants of the UK Biobank needed for our validation and testing strategy [32,33]. The summary statistics were used as input into the LDPred computational algorithm, which reweights each variant according to its effect size, strength of statistical significance, correlation with nearby variants, and a global tuning parameter that denotes the number of variants with non-zero effect size [29]. Because the optimal value of this global tuning parameter is difficult to know *a priori*, a range of six values was tested as previously recommended in order to create six candidate scores [29].

To select the global tuning parameter, we assessed our candidate scores in an independent validation set of 119,248 randomly-selected participants of European ancestry from the UK Biobank of whom 279 (0.2%) had been diagnosed with Alzheimer’s disease. Each of the 6 candidate scores was associated with disease in logistic regression models that included age, sex, and principal components of ancestry as covariates. Odds ratios per standard deviation higher polygenic score in these models ranged from 1.1 to 1.9 and area under the receiver operator curve (AUROC) ranged from 0.72 to 0.78 (**S1 Table**).We selected the score with the maximal AUROC (0.78) to carry forward into our testing set of 288,940 additional UK Biobank participants, all of whom were distinct from our validation set. Among these participants, mean age at enrollment was 57 years, 54% were female, and 651 (0.2%) had been diagnosed with Alzheimer’s disease. Results in the testing dataset were highly concordant with the validation dataset, with odds ratio per standard deviation higher polygenic score of 1.9 (95% CI 1.7 to 2.0; p = 4.6 x 10^−69^) and AUROC of 0.77, accounting for 3.4% of the observed variance. We estimate that 64% of this variance explained was contributed by variants near the gene encoding apolipoprotein E (*APOE*)–which include the well-known ApoE ε4 risk haplotype [34–36]–and 36% by variants in the remainder of the genome (see Methods). This model was well calibrated (calibration slope: 1.04; Hosmer-Lemeshow p value: 0.19; **S1 Fig**). As expected, the frequency of the ApoE ε4 risk haplotype varied substantially across polygenic score deciles–from an allele frequency of 0 for those in the lowest decile to 59% for those in the highest decile (**S2 Fig**).

The association between polygenic score for Alzheimer’s disease and disease was analyzed in a testing set of 288,940 UK Biobank participants, of whom 651 had been diagnosed with Alzheimer’s disease. Odds ratios were calculated by comparing those with high polygenic score to the middle quintile of the population in a logistic regression model adjusted for age, sex, genotyping array, and the first four principal components of ancestry.

**Fig 2:**
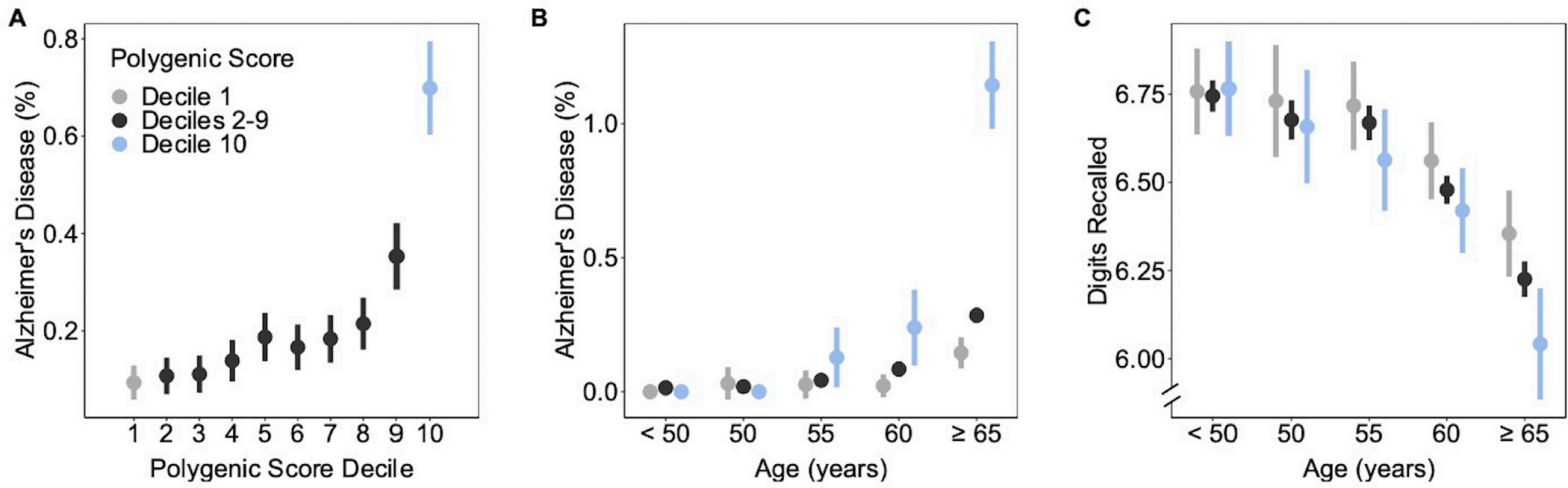
Association of a polygenic score for Alzheimer’s disease with clinical diagnosis and cognitive function. **a**. Relationship of polygenic score decile to rates of Alzheimer’s disease diagnosis within the UK Biobank testing dataset. **b**. Age-stratified analysis of the relationship between polygenic score decile groupings and Alzheimer’s disease diagnosis within the UK Biobank testing dataset. Age is assigned based on age at diagnosis of Alzheimer’s disease for those affected or date of last follow-up for others. **c.** Age-stratified analysis of the relationship between polygenic score decile groupings and performance on a ‘digit recall test,’ a measure of cognitive function. Age is binned into groups corresponding to <50, ≥50–54, ≥55–59, ≥60–64, and ≥65 years at time of assessment. Error bars represent 95% confidence intervals.

Across the entire testing dataset, presence of Alzheimer’s disease ranged from 0.1% in the bottom decile to 0.7% in the top decile, corresponding to an adjusted odds ratio of 7.3 (95% CI 4.8 to 11.0; p = 4.5 x 10^−21^; **Fig 2A**). As noted for other diseases, increased risk was most pronounced for those in the extreme tail of the distribution [23–25]. As compared to those in the middle quintile, odds ratios for those in top 20%, 10%, 5%, and 1% of the score distribution were 3.1, 4.2, 5.1, and 6.2 respectively (**Table 1**).

**Table 1:** Association of High Polygenic Score with Alzheimer’s Disease in the UK Biobank.

High Polygenic Score Definition	Reference Group	Odds Ratio for High Polygenic Score (95% CI)	P Value
Top 1% of distribution	Middle Quintile	6.2 (4.1–9.2)	5.9 x 10^−19^
Top 5% of distribution	Middle Quintile	5.1 (3.6–7.2)	8.1 x 10^−20^
Top 10% of distribution	Middle Quintile	4.2 (3.0–5.8)	2.0 x 10^−17^
Top 20% of distribution	Middle Quintile	3.1 (2.3–4.3)	2.4 x 10^−12^

### Age dependent association of Alzheimer’s disease polygenic score with Alzheimer’s disease

Given that rates of Alzheimer’s disease are known to increase substantially with age, we next performed age-stratified analyses (**Fig 2B**). Among participants aged less than 50 years, almost none had been diagnosed with disease and there was no detectable gradient according to polygenic score (0% in the bottom decile, 0.01% for those in deciles 2–9 and 0% in top decile, p = 0.45). However, with increasing age, we noted progressively more pronounced gradients. Among individuals aged 65 years and older, the gradient had increased significantly– 0.1% versus 1.1% for those in the bottom versus top decile, respectively (p = 4.5 x 10^−21^). We replicated this age-dependent association of the polygenic score with Alzheimer’s disease among 30,541 participants of the Mass General Brigham Biobank, of whom 460 (1.5%) had a diagnosis of Alzheimer’s disease (**S3 Fig**). We again noted no significant differences at younger ages, but for those over 65 years we noted a prevalence of 2.0% versus 4.0% in the bottom versus top decile respectively, p = 0.002.

### Alzheimer’s disease polygenic score is associated with cognitive function

Because a clinical diagnosis of overt Alzheimer’s disease occurs late in the disease process, we explored the existence of similar variability in disease trajectory using a subclinical measure of cognitive function. Among 30,853 participants with available genetic data who completed the assessment, mean number of digits recalled was 6.5 (standard deviation 1.7). As noted for disease diagnoses, we noted no significant difference for those less than 45 years but progressively larger differences among older participants (**Fig 2C**). For those aged 65 years or older, the mean number of digits remembered was 6.4 versus 6.0 digits among those in the bottom versus top decile respectively, p = 0.002). Results were nearly identical in a sensitivity analysis that removed 65 participants who had been previously diagnosed with Alzheimer’s disease.

### A high polygenic score is associated with circulating proteins in asymptomatic individuals

Polygenic risk scores have important potential implications for biomarker discovery because they identify at-risk individuals before they experience symptoms. To test the hypothesis that circulating biomarkers would vary according to polygenic risk for Alzheimer’s disease among putatively unaffected individuals, we studied 3,231 circulating proteins using the Somalogic aptamer-based assay in the INTERVAL study of 3,175 blood donors in the UK [37,38]. We compared levels of each of the proteins for those in the bottom versus top decile of the polygenic score (n = 318 in each group). Among these 636 participants, mean age was 43 years and 47% were female without significant differences in age or sex according to the polygenic score (**S2 Table**).

**Fig 3:**
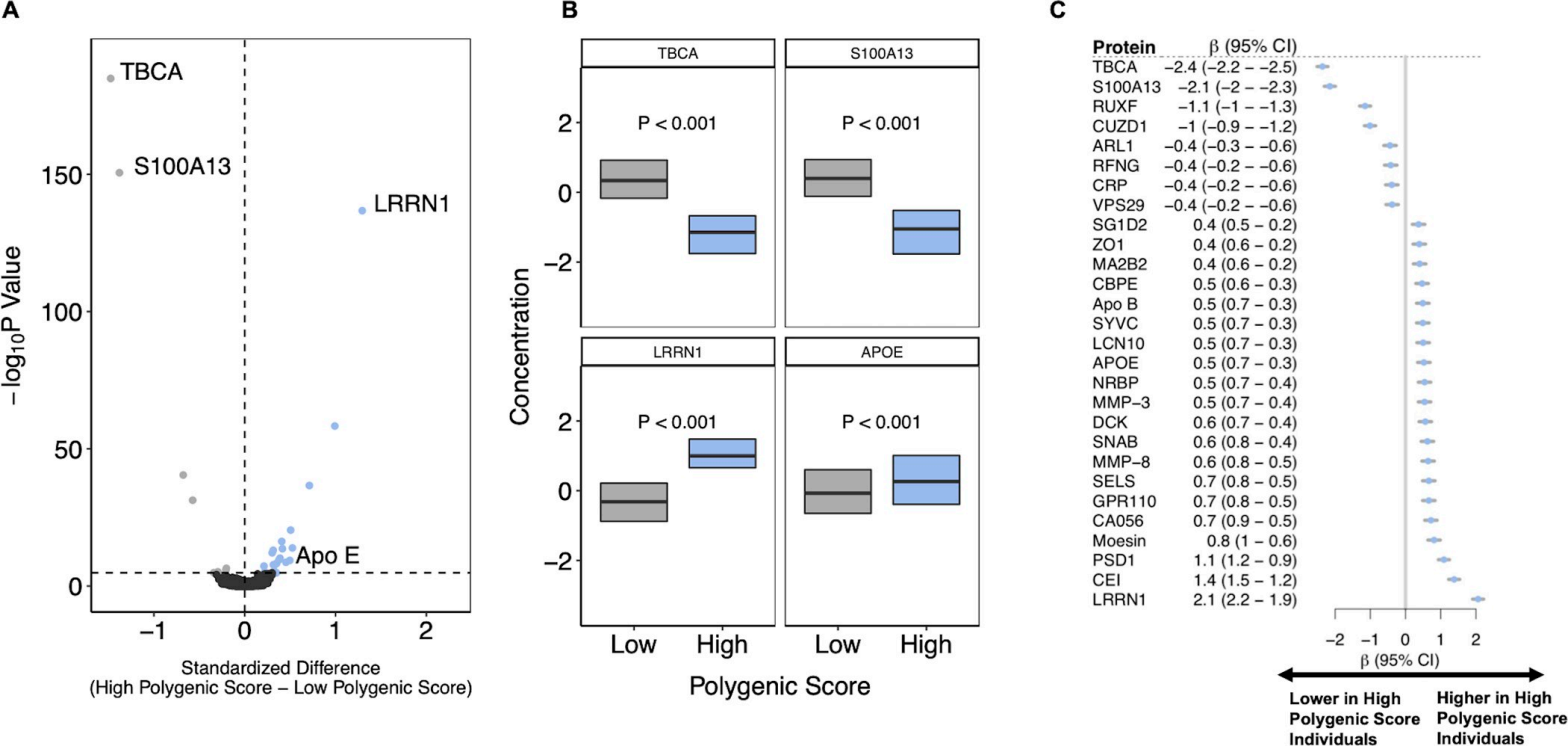
Proteomic signature of inherited risk for Alzheimer’s disease a. The levels of each of 3,231 plasma proteins quantified using an aptamer-based assay were compared between 636 participants from the INTERVAL study with top versus bottom decile of the polygenic score in models adjusted for age, sex, duration between blood draw and processing and the first three principal components of ancestry. The x-axis shows difference–in standardized units with mean 0 and standard deviation 1 –in concentration and the y-axis -log_10_ p-value for strength of association. The horizontal dashed line represents the Bonferroni-corrected threshold for statistical significance (*P* < 1.55 x 10^−5^). **b.** Boxplots show levels of the three most significantly associated proteins and apolipoprotein E, a known Alzheimer’s disease-related protein. **c.** The associations between 28 proteins with levels that significantly differed according to high vs low polygenic score. The x-axis refers to the difference in concentration in standardized units. Whiskers represent 1.5*IQR. TBCA: tubulin-specific chaperon protein A; S100A13: S100 calcium-binding protein A13; RUXF: Small Nuclear Ribonucleoprotein Polypeptide F; CUZD1: CUB and zona pellucida-like domain-containing protein 1; ARL1: ADP-ribosylation factor-like protein 1; CRP: C-reactive protein; VPS29: Vacuolar protein sorting-associated protein 29; SG1D2: Secretoglobin Family 1D Member 2; ZO1: Tight junction protein 1; MA2B2: Mannosidase Alpha Class 2B Member 2; CPBE: Choline binding protein E; ApoB: Apolipoprotein B; SYVC: Valyl-TRNA Synthetase 1; LCN10: Lipocalin 10; APOE: Apolipoprotein E; NRBP: Nuclear Receptor Binding Protein 1; MMP-3: matrix metalloproteinase-3; DCK: Deoxycytidine kinase; SNAB: Beta-soluble NSF attachment protein; MMP-8: matrix metalloproteinase-8; SELS: Selenoprotein S; GPR110: Adhesion G-protein coupled receptor F1; CA056: Protein MENT; PSD1: PH and SEC7 domain-containing protein 1; CEI: Protein CEI; LRRN1: Leucine-rich repeat neuronal protein 1

Given a well-characterized role in amyloid plaque deposition [39–41], levels of apolipoprotein E served as a useful positive control. We noted significantly increased levels of apolipoprotein E in participants with a high polygenic score, mean values (expressed in terms of Z score as described previously) of -0.05 versus 0.28 for those in bottom versus top decile respectively (p = 2.3 x 10^−9^; **Fig 3A and 3C**) [37].

In addition to apolipoprotein E, there were 27 additional proteins whose levels varied according to low versus high polygenic score for Alzheimer’s disease at a Bonferroni corrected p-value 1.5 x 10^−5^ (0.05/ 3231; **Fig 3A and 3C and S3 Table**). For several proteins, the differences in levels were significantly more pronounced than for apolipoprotein E. The strongest associated biomarker was tubulin specific chaperone A (**Fig 3B**), a protein with a role in preventing neurotoxicity due to abnormal beta tubulin folding.^42^ Individuals with a high polygenic score had substantially lower circulating levels of this protein–mean score of 0.40 versus -1.2 for those in bottom versus top decile. For other proteins, such as S100 calcium binding protein A13 (a member of the S100 family known to interact with the advanced glycation end product pathway [42,43]) and leucine-rich repeat neuronal protein (known to regulate early neuronal progenitor cell signaling [44]), levels were substantially higher in those with higher inherited risk. Additional description of each of the 28 polygenic score-associated proteins is presented in **S4 Table**).

Among the 28 proteins associated with a high polygenic score, 20 proteins had at least one cis-pQTL or trans-pQTL in the INTERVAL cohort, consisting of 14 unique pQTLs. Several of the pQTLs were in known AD-risk genes including APOE, APOC4, APOC1, C7, CRP (**S5 Table**). Among the 14 pQTLs, 7 were significantly associated with the overall polygenic score.

As an additional sensitivity analysis, we restricted our proteomics analysis to younger participants from the INTERVAL study, in whom any meaningful clinical manifestation of Alzheimer’s disease is even less likely to have occurred. Among 334 participants aged less than 45 years (mean 33 years)– 163 with a polygenic score in the bottom decile versus 171 in the top decile–we note directionally consistent and nominally significant results (p <0.05 in a logistic model that included age, sex and the first four principal components of ancestry) for 25 out 28 proteins identified in the overall cohort (**S4 Fig**).

To assess the generalizability of our results to a multiethnic population, we computed the association between polygenic score and each of the 28 proteins in the multi-ethnic MESA cohort [31]. Of the 28 proteins associated with a high polygenic score in INTERVAL, 8 were measured in the MESA study: SNRPF, Moesin, MMP8, MMP3, APOE, APOB, CBPE, and CRP. We compared levels of each of the proteins for those in the bottom versus top decile of the polygenic score (n = 170 in each group). Among these 340 participants, mean age was 60 years and 53% were female. Seven proteins (all except MMP3), were also associated with a high versus low polygenic score in MESA (**S5 Fig**).

## Discussion

In this study, we describe a systematic approach to identify a proteomic signature of an elevated genetic susceptibility to disease quantified through a polygenic score. Focusing on Alzheimer’s disease as a common disease with significant public health burden for which few circulating biomarkers exist, we first computed a polygenic score using previously published summary association statistics. In an independent testing cohort from the UK Biobank, we found a striking association between the polygenic score and diagnosis of Alzheimer’s disease and cognitive function, a finding that was replicated in the independent Mass General Brigham biobank. Interestingly, we found that an elevated polygenic score for Alzheimer’s disease is associated with levels of 28 circulating proteins in a group of 636 healthy, middle aged participants in the INTERVAL cohort. For 25 out of the 28 proteins, their association with a high polygenic score was present even among individuals <45 years of age, suggesting an early proteomic signature of disease that begins decades before clinical manifestation of Alzheimer’s disease.

Our analysis of the relationship between a polygenic score for Alzheimer’s disease with disease trajectories and potential new biomarkers has at least two implications:

First, one possible reason for failure of past Alzheimer’s trial may be intervention too late in the disease process [42]. These failures–which are costly and likely to have prevented additional investment in drug development–often occur even when a therapeutic target is believed to be pathophysiologically sound, as was the case for solanezumab, an antibody designed to clear amyloid-beta from the brain.^47,48^ While there have been examples of clinical trials aimed at rare genetic forms of early-onset Alzheimer’s disease [45–47], a primary prevention trial enrichment strategy focused on middle-aged asymptomatic individuals with high polygenic score might prove useful [48].

Second, molecular profiling of individuals with very high or very low inherited risk based on a polygenic score–but who remain unaffected–may provide a new approach to nominating new biomarkers or pathways for a given disease [38]. This strategy is different from the traditional approach of profiling individuals after symptom onset, where distinguishing whether changes are a cause or consequence of disease onset often proves challenging. Although differences in circulating biomarkers do not prove disease relevance, additional research into those nominated here may prove useful in uncovering new biology or serving as biomarkers of therapeutic efficacy or target engagement within drug development efforts.

In the current study, our finding that levels of APOE were increased in individuals with a high polygenic score served as a useful positive control, given the well-documented role of APOE in the pathophysiology of Alzheimer’s disease. Serum levels of APOE have been associated with increased risk of developing Alzheimer’s disease and cognitive impairment [49,50]. In addition to proteins known to play a pathophysiological role in Alzheimer’s disease such as APOE, numerous other proteins were associated with the polygenic score and replicated in the MESA cohort. Overall, we found 8 proteins whose levels were lower in the high polygenic score group and 20 proteins whose levels were higher in the high polygenic score group. Among the proteins whose levels were lower in the high polygenic score were a number of proteins critical for maintaining the integrity of endolysosomal-trans-golgi axis, an important mechanism for neuronal proteostasis [51]. For example, VPS29 is one such protein that is part of the retromer complex which functions in recycling protein cargoes from endosomes to the trans-golgi network. This process has been associated with amyloid beta trafficking and processing, and deficiency in retromer has been associated with neuronal loss and amyloid-beta aggregation in a mouse model of Alzheimer’s [52]. Another protein whose levels were lower the high polygenic score group is Arl1, whose downregulation leads to loss of trans-golgi cisternae [53]. Overall, these findings support the hypothesis of an early defect in the endolysosomal-trans-golgi network priming the brain for amyloid-beta accumulation. Among the proteins elevated in the high polygenic score group include MMP-8 and MMP-3, members of the metalloproteinase family. MMP-8 is known to play a role in macrophage [54] and microglia-mediated immune activation [55]. These results suggest a role for increased peripheral and central nervous system immune activation in Alzheimer’s disease, a finding that has been observed by others and validated through PET neuroimaging [56,57] and CSF studies [58–60]. Further, MMP-8 has been widely nominated as a therapeutic target in AD [61,62], suggesting the ability of proteomic profiling at the extremes of a polygenic score distribution to uncover therapeutic targets. Interestingly, other than APOE, none of the genes encoding the 28 polygenic score-associated proteins are near (<500kb) loci implicated in Alzheimer’s disease GWAS efforts [63]. This suggests the proteins identified using our approach would likely not have been identified in traditional GWAS studies.

Several limitations exist to the current study. Although we demonstrate here–and others have demonstrated previously [64–68]–that it is possible to create a polygenic score for Alzheimer’s disease, we urge caution prior to deployment outside of a research setting. First, as is the case with most polygenic scores developed to date, effect size is likely to be lower in non-European populations due to lack of training data [67,69]. Second, current clinical guidelines do not yet support assessment of genetic risk for Alzheimer’s’s disease outside of suspected rare monogenic forms, largely due to concerns about implications for long-term-care or disability insurance, inducing anxiety, and relative absence of efficacious preventive measures [64]. The polygenic score developed in the present study demonstrated an odds ratio per standard deviation increase of 1.90. Although this effect estimate is comparable to that noted with other recent polygenic scores [64–67]–with odds ratios per standard deviation increase ranging from 1.38 to 2.20–we did not directly compare them in the present study. Additional efforts to characterize the relationship between future polygenic scores, neurocognitive trajectory, and proteomic signatures are warranted in future studies. Additionally, several rare mutations of large effect have been associated with Alzheimer’s disease [17–21], our polygenic score was restricted to common DNA variants. Future efforts to develop an integrated risk model that includes both common and rare variants for Alzheimer’s disease is likely to be of significant utility. Another limitation of the current study is the lack of a multiethnic polygenic score, which is important given the reduction in performance when European-derived scores are applied to non-European populations [19,70,71]. A key additional limitation of the current study is limitation of the analysis to individuals of European ancestry. While these analysis provide important proof-of-concept for the potential value of polygenic scoring for risk stratification or clinical development, additional assessment in diverse ancestral populations or development of a multiethnic polygenic score are of major interest. Lastly, while we replicated proteins associated with a high versus low polygenic score in the MESA cohort, additional replication in large-scale studies will be of interest.

## Methods

### Ethics statement

This research was approved by the UK Biobank Application Committee (application number 7089) and by the Massachusetts General Hospital Institutional Review Board.

### Informed consent and study approval

All participants provided written informed consent at the time of enrolling in the UK Biobank, INTERVAL, MESA and Mass General Brigham Biobank studies. Analysis for this study was approved by the Mass General Brigham Institutional Review Board (Boston, MA).

### Study cohorts

The polygenic score was validated and tested in the UK Biobank, a large observational, longitudinal study that enrolled 502,505 participants aged 40–69 from centers across the United Kingdom starting in 2006[70]. A subset of participants completed a cognitive assessment, including the Forward Digit Span Test to assess working memory [71]. We selected participants who underwent genomic profiling using either of two genotyping arrays covering 800,000 common genetic markers [29]. Genotype imputation was performed previously by the UK Biobank using the Haplotype Reference Consortium panel version 1.1, the UK10K panel, and the 1000 Genomes panel. To minimize potential confounding related to genetic ancestry, analyses were restricted to participants of White British ancestry previously defined by the UK Biobank using a combination of self-reported ancestry and genetic confirmation. Quality control was performed as described previously [29]. In brief, participants were excluded based on quality control metrics, previously computed by the UK Biobank, including a high genotype missing rate, sex discordance, putative sex chromosome aneuploidy, and withdrawal of informed consent.

Within the UK Biobank, participants with Alzheimer’s disease were identified centrally using a combination of primary care, patient inpatient hospital records, and mortality records using the International Classification of Disease (ICD-10) diagnosis code of G30 and READ code F00 (UK Biobank Field ID 131036).

The INTERVAL BioResource involves ~50,000 blood donors recruited from 25 centres across England during 2012–2014[30]. Study enrollment criteria were consistent with standard blood donation criteria defined by National Health Service Blood and Transplant [72] and excluded individuals with history of major disease including heart disease, stroke, diabetes, atrial fibrillation, type 2 diabetes requiring medications, cancer and recent illness or infection [30,73]. Genotyping was performed using the Axiom UK Biobank genotyping array developed by Affymetrix (Santa Clara, California, US). Sample and variant quality control had been performed previously and involved exclusion based on sex mismatch, low genotype call rates, duplicate samples, extreme heterozygosity and non-European ancestry, as described earlier [37]. Genotyping imputation was performed previously [37] using the UK10K and 1000 Genomes reference panels.

The polygenic score was independently tested in a cohort of 30,541 European-ancestry participants of the Mass General Brigham Biobank who had previously undergone genomic profiling [74]. Among this cohort, 458 participants had been diagnosed with Alzheimer’s disease based on inclusion of the ICD-10 code G30.X in the electronic health record. Age of Alzheimer’s disease diagnosis or last follow-up for controls, sex and the first four principal components of ancestry were recorded for each participant. Samples were imputed to the Haplotype Reference Consortium panel version 1.1 using the Michigan Imputation Server [27,75].

Among the 45,263 blood donors originally recruited in the INTERVAL cohort, 3,562 underwent proteomic profiling in two batches using 4,034 SOMAscan aptamers developed by SomaLogic Inc. (Boulder, Colorado, US) as previously described [37]. In brief, the SOMAscan technology allows for the simultaneous measurement of thousands of proteins from small sample volumes (15 uL serum or plasma) with a lower detection limit compared to traditional methods such as immunoassays [76,77]. The SOMAscan aptamer panel measures both intracellular and extracellular proteins with a bias towards secreted proteins, reflecting the availability of purified protein targets and targets with a putative role in human disease [76,77].

The Multi-Ethnic Study of Atherosclerosis (MESA) cohort was used to replicate proteins significantly associated with a high versus low polygenic score. The design of the MESA study has been described previously and the protocol is available at www.mesa-nhlbi.org. In brief, MESA is a multiethnic prospective cohort that enrolled 6,814 participants in the United States free of cardiovascular disease between 2000 and 2002[31]. Whole genome sequencing was performed on a subset of 3,932 participants, of whom 3,761 were retained after application of sample and variant quality control criteria, as described previously [69].

### Polygenic score derivation and validation

Polygenic scores quantify genetic risk across common variants (minor allele frequency ≥1%) by summing variants weighted by the strength of their association with a given trait. To derive a polygenic score for Alzheimer’s disease, we first divided the UK Biobank into a validation set of 119,248 participants and a test set of 288,940 non-overlapping participants. Within the validation set, we used the LDPred computational algorithm, summary statistics from a recent genome-wide association study for Alzheimer’s disease [26] and a reference panel of 503 European-ancestry participants from 1000 Genomes phase 3 version 5[27] to derive candidate polygenic scores.

The LDPred algorithm uses a Bayesian approach to calculate posterior mean effect sizes using genome wide association summary statistics by assuming priors for genetic architecture and linkage disequilibrium from a reference panel. A tuning parameter, ρ, is used to control the fraction of causal (ie. non-zero effect size) variants. Consistent with previous work [23], a range of tuning parameters– 1, 0.3, 0.1, 0.03, 0.01, 0.003 –was used to derive 6 candidate polygenic scores. Each candidate polygenic score was calculated in the validation set by multiplying the genotype dosage of each risk allele by its respective variant weight, and then summing across all variants in the score using PLINK2^79^ software, as previously described [23]. To account for subtle variation in genetic ancestry that may confound the association between polygenic score and Alzheimer’s disease, we corrected our polygenic score for the effects of ancestry as described previously [23]. In brief, a linear regression model was used to predict polygenic score using the first four principal components of ancestry. The residual from this model was retained as an ancestry-corrected polygenic score for downstream analysis

The polygenic score with the best discriminative capacity was defined as the score with the maximal AUROC in a logistic regression model with Alzheimer’s disease as the outcome and the candidate ancestry-corrected polygenic score, age, sex, first four principal components of ancestry. The best polygenic score was applied to the test set.

### Assessment of polygenic score in the UK Biobank test set

Within the UK Biobank testing dataset, we first assessed the risk of Alzheimer’s disease for participants in the top 1%, top 5%, top 10% and top 20% of the polygenic score distribution compared to those in the middle quintile. A logistic regression model was fit using covariates of an indicator variable for having a top polygenic score vs middle quintile score, age, sex, and the first four principal components of ancestry and Alzheimer’s disease as the outcome. For each model, we calculated the odds ratio conferred by having a high polygenic score.

To determine the relative contribution of variants near the *APOE* gene region to the predictive ability of our polygenic score in the UK Biobank testing dataset, we compared the proportion of variance explained–using the Nagelkerke’s pseudo-R^2^ metric–for two models: (i) a base logistic regression model that included only the covariates of age, sex, and the first four principal components of ancestry and (ii) the covariates plus the polygenic score.

We assessed the gradient in Alzheimer’s disease prevalence across polygenic score deciles. Individuals in the test set were split into polygenic score deciles and disease prevalence was calculated. An odds ratio for the top decile vs bottom decile was calculated using a logistic regression model with Alzheimer’s disease as the outcome and age, sex, and the first four principal components of ancestry as covariates. Calibration curves and intercepts were derived by fitting a linear regression model with observed Alzheimer’s prevalence as the outcome variable and predicted prevalence as the independent variable. Goodness of fit was evaluated using the Hosmer-Lemeshow test.

Age-stratified analyses were conducted by dividing the test set into age groups corresponding to <50, ≥50–54, ≥55–59, ≥60–64, and ≥65 years. Age was assigned based on age at diagnosis of Alzheimer’s disease for those affected or date of last follow-up for others based on the most recent available hospital inpatient record, mortality record, or primary care re cord. Participants were also characterized as belonging to the bottom decile, deciles 2–9, or top decile of polygenic score. For each age category, we compared the prevalence of Alzheimer’s disease among participants in the bottom decile to those in the top decile using a logistic regression model adjusted for sex and the first four principal components of ancestry.

To assess the association between Alzheimer’s disease polygenic score and working memory, we analyzed 30,853 participants who underwent cognitive testing in the UK Biobank. As part of the study protocol, UK Biobank participants completed a test of numeric short-term memory based on ability to recall strings of digits of various length (‘digit span test’) [71]. Polygenic score was associated with the number of digits recalled on the Digit Span Test using a linear regression model that included age, sex, and the first four principal components of ancestry as covariates. A sensitivity analysis conducted by removing participants diagnosed with Alzheimer’s disease yielded nearly identical results.

All statistical analyses were conducted using R version 3.6.1 (The R Foundation).

### Assessment of polygenic score in the Mass General Brigham Healthcare Biobank

The age-dependent association between polygenic score and Alzheimer’s disease was independently tested in the Mass General Brigham Biobank [74]. As in the UK Biobank, the Mass General Brigham cohort was divided into age groups corresponding to <50, 50–54, 55–59, 60–64, and ≥65 years. Participants were also characterized as belonging to the bottom decile, middle 2nd-9th deciles, or top deciles of polygenic score. For each age category, we compared the prevalence of Alzheimer’s disease among participants in the bottom decile to those in the top decile using a logistic regression model with sex and first four principal components of ancestry as covariates.

### Assessment for a proteomic signature of high versus low polygenic score

For participants in the INTERVAL cohort who underwent proteomic profiling, data processing and quality control were performed as described previously [30]. A multiplexed, aptamer-based approach (SomaLogic SOMAscan assay) was used to measure the relative levels of 3,622 plasma proteins or protein complexes, using 4,034 modified aptamers. Assayed proteins were selected based on the availability of purified protein targets, and screening of proteins that are likely to be involved in human disease. Quality control metrics for the SOMAscan platform have been described [30]. When multiple aptamers mapped to the same protein, we selected the aptamer with strongest binding affinity (K_d_) measured using pulldown pull-down assays followed by mass spectrometry and SDS-based gel to assess the binding affinity of each SOMAmer for its target, as described.^82^ Following quality control, 3,231 proteins were retained for analysis.

To test the associations of plasma protein levels with a high polygenic score for Alzheimer’s disease, we first natural log-transformed the relative protein abundances. Log-transformed protein levels were then adjusted in a linear regression model for age, sex, duration between blood draw and processing (binary, ≤1 day/>1day) and the first three principal components of ancestry as described previously [37]. The protein residuals from this linear regression were then rank-inverse normalized and used as phenotypes for association testing. Participants in the INTERVAL cohort were dichotomized as belonging to the top polygenic score decile (high polygenic score) or bottom polygenic score decile (low polygenic score), the genotype dosage of each risk allele was multiplied by its respective variant weight, and then summed across all variants to yield a score using PLINK2[28] software. Adjusted protein levels were compared between high and low polygenic score participants using a two-sample t-test. A p value < 1.55 x 10^−5 (^0.05/3231) was deemed significant. A sensitivity analysis was conducted by restricting analysis to participants < 45 years of age at the time of plasma sampling.

Protein quantitative trait loci (pQTL) were identified for proteins significantly associated with the polygenic score. pQTLs were obtained using previously published summary statistics from the INTERVAL cohort [37]. Genetic associations were considered significant using a genome-wide threshold as previously described [37]. The association between pQTLs and AD PRS was examined using a linear regression model with AD PRS as the outcome and pQTL, age, sex, and principal components as covariates.

### Replication of proteomic markers of proteomics signature of high versus low polygenic score in the MESA cohort

A subset of MESA participants underwent proteomic profiling using an older version of the SOMAscan platform–including 1,319 markers–using samples obtained at Exam 1 (2000–2002) as previously described [76]. Following quality control, 846 individuals who underwent both proteomic profiling and whole genome sequencing profiling were available for analysis. This cohort self-identified as White (n = 742, 44%), Asian (n = 108, 6%), Black (n = 338, 20%) and Hispanic (n = 512, 30%). To compute the AD polygenic score for Alzheimer’s disease in MESA, the genotype dosage of each risk allele was multiplied by its respective variant weight, and then summed across all variants to yield a score using PLINK [78]. To enable analysis across the four self-reported MESA ethnic/racial groups, an ancestry-corrected polygenic score was computed by retaining the residuals of a linear regression model in which the polygenic score was regressed against the first three principal components of ancestry. Participants in the MESA cohort were dichotomized as belonging to the top ancestry-corrected polygenic score decile (high polygenic score; n = 85) or bottom ancestry-corrected polygenic score decile (low polygenic score, n = 85).

For the subset of protein markers that were available in the MESA study participants, we sought to replicate results from the INTERVAL study. Relative protein abundances were first natural log-transformed. Log-transformed protein levels were then adjusted in a linear regression model for age, sex, and the first three principal components of ancestry. The protein residuals from this linear regression were then rank-inverse normalized and used as phenotypes for association testing. Adjusted protein levels were compared between high and low polygenic score individuals using a two-sample t-test. A nominal one-tailed p-value < 0.05 with the direction of effect prespecified based on the INTERVAL analysis was deemed statistically significant.

## Supporting information

S1 Fig Calibration plots in the testing cohort. A logistic regression model that included the AD PRS, age, sex, and principal components of ancestry as covariates was well-calibrated in the test dataset. Slope of the calibration curve is displayed. Error bars represent 95% CI.
(DOCX)

S2 Fig Distribution of the APOE ε4 allele among polygenic score deciles. The distribution of APOE ε4 is presented for each polygenic score decile, ranging from 0.59 APOE ε4 allele frequency in the top decile to 0 in the bottom decile. Consistent with the 64% contribution of variants near the gene encoding apolipoprotein E (*APOE*) to the polygenic score, we observe significantly more APOE ε4/ε4 homozygous individuals in the top polygenic score decile (23%) compared to the bottom (0%).
(DOCX)

S3 Fig Age-stratified relationship between polygenic score and Alzheimer’s disease diagnosis in the Mass General Brigham Biobank. The Alzheimer’s disease polygenic score was independently validated in the Mass General Brigham Biobank. Age was assigned based on age at diagnosis of Alzheimer’s disease for those affected or date of last follow-up for others. Similar to the UK Biobank, we observe a significant gradient in Alzheimer’s disease prevalence across polygenic score deciles at later ages in a logistic regression model adjusted for sex and the first four genetic principal components. Error bars represent 95% confidence intervals.
(DOCX)

S4 Fig Sensitivity analysis of circulating protein levels and polygenic score in individuals < 45 years. To assess differences in protein levels among individuals <45 years (mean 32.6 years), when the onset of Alzheimer’s disease is even more unlikely, we analyzed standardized levels of the 28 proteins identified in the overall dataset. A low polygenic score indicates individuals in the first decile of the distribution and a high score indicates individuals tenth decile. * represent proteins with levels significantly different between high and low polygenic score individuals. In middle age, protein levels are consistently associated with polygenic score (p<0.05, two-tailed t-test). Whiskers represent 1.5*IQR.
(DOCX)

S5 Fig Replication of proteomic signature of high polygenic score in the MESA cohort. Boxplots are displayed comparing levels of 8 proteins in individuals with a high polygenic score for Alzheimer’s disease (top 10%) and a low polygenic score (bottom 10%) in the MESA cohort. Of the 28 proteins associated with a high polygenic score in the INTERVAL discovery cohort, 8 proteins were available in the MESA cohort. Among the 8 proteins assayed, 7 replicated their association with a high polygenic score for Alzheimer’s disease. P values computed using a two-sample one-tailed t-test using adjusted protein levels (see Methods). Whiskers represent 1.5*IQR.
(DOCX)

S1 Table Association of candidate polygenic scores with Alzheimer’s Disease in UK Biobank validation set. To select the global tuning parameter, six candidate scores were assessed in a validation set of 119,248 randomly-selected participants of European ancestry from the UK Biobank of whom 279 (0.2%) had been diagnosed with Alzheimer’s disease. Each candidate score was associated with disease in logistic regression models that included age, sex, and principal components of ancestry as covariates and odds ratio (OR) per standard deviation (SD) of polygenic score and area under the receiver operator curve (AUROC) was calculated. The tuning parameter refers to the LDpred ρ parameter used to control the proportion of variants assumed to be causal. Bold indicates polygenic score with maximal AUROC carried forward to the testing datasets. The calibration curves and intercepts were derived by fitting a linear regression model with observed Alzheimer’s prevalence as the outcome variable and predicted prevalence as the independent variable.
(XLSX)

S2 Table INTERVAL cohort characteristics. *P value defined using a two-sample t-test or Chi-squared test for categorical variables.
(XLSX)

S3 Table AD Polygenic Score-Protein Associations. Beta represents average change in protein level among individuals in 90% AD PRS compared to those in the 10%.
(XLSX)

S4 Table Description and evidence for role in Alzheimer’s disease of each polygenic score-associated protein. (XLSX)

S5 Table Proteins with pQTL variants and their association with AD PRS. pQTL- AD PRS assocation was ascertained in a linear regression model with AD PRS as the outcome and pQTL, age, sex, and principal components as covariates. Beta represents the average change in AD PRS for a 1 unit change in pQTL variant where the pQTL variant is encoded as 0,1,2. A P value < 0.05/14, where 14 is the number of unique pQTL variants, was considered significant. pQTL variants within 1Mb of an aptamer were considered as cis-pQTL with remaining variants being trans-pQTLs. A P value < 0.05/14, where 14 is the number of unique pQTL variants considered, was considered significant.”
(XLSX)
