# Supplementary material for: Neurocognitive trajectory and proteomic signature of inherited risk for Alzheimer’s disease
Source: PLoS Genet. 2022 Sep 1;18(9):e1010294. doi: 10.1371/journal.pgen.1010294 (PMC9436054; doi:10.1371/journal.pgen.1010294)
Supplement: S1 Fig — A logistic regression model that included the AD PRS, age, sex, and principal components of ancestry as covariates was well-calibrated in the test dataset. Slope of the calibration curve is displayed. Error bars represent 95% CI. (DOCX) [file pgen.1010294.s001.docx]

**Figure S1: Calibration plots in testing cohort**

**
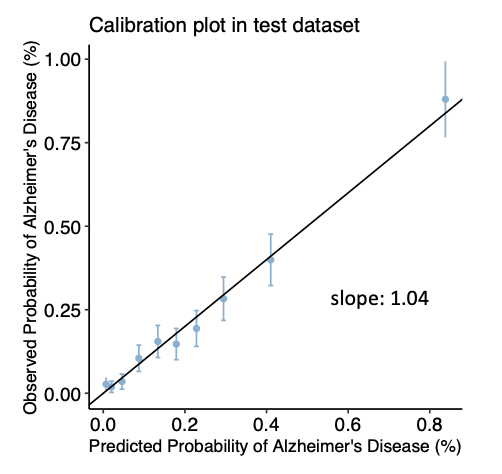
**

A logistic regression model that included the AD PRS, age, sex, and principal components of ancestry as covariates was well-calibrated in the test dataset (slope: 1.05, intercept: 9.9 x 10^-5^, Hosmer-Lemeshow P value: 0.19).  Error bars represent 95% CI.
