# Supplementary material for: Neurocognitive trajectory and proteomic signature of inherited risk for Alzheimer’s disease
Source: PLoS Genet. 2022 Sep 1;18(9):e1010294. doi: 10.1371/journal.pgen.1010294 (PMC9436054; doi:10.1371/journal.pgen.1010294)
Supplement: S2 Fig — The distribution of APOE ε4 is presented for each polygenic score decile, ranging from 0.59 APOE ε4 allele frequency in the top decile to 0 in the bottom decile. Consistent with the 64% contribution of variants near the gene encoding apolipoprotein E (APOE) to the polygenic score, we observe significantly more APOE ε4/ε4 homozygous individuals in the top polygenic score decile (23%) compared to the bottom (0%). (DOCX) [file pgen.1010294.s002.docx]

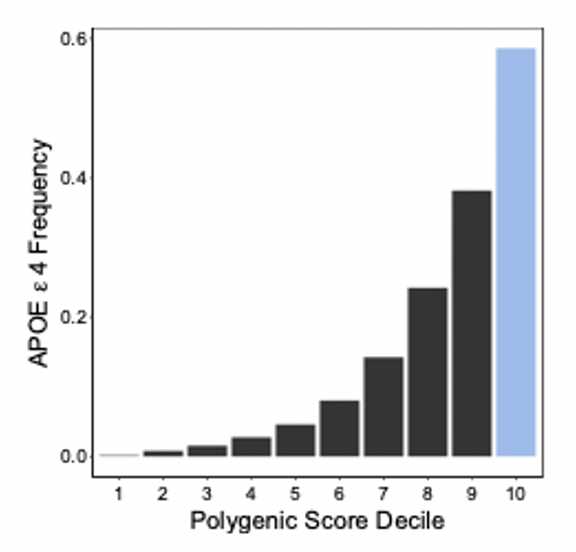
**FIGURE S2: Distribution of the APOE ε4 allele among polygenic score deciles.**

The distribution of APOE ε4 is presented for each polygenic score decile, ranging from 0.59 APOE ε4 allele frequency in the top decile to 0 in the bottom decile. Consistent with the 64% contribution of variants near the gene encoding apolipoprotein E (*APOE*) to the polygenic score, we observe significantly more APOE ε4/ε4 homozygous individuals in the top polygenic score decile (23%) compared to the bottom (0%).
