# Supplementary material for: Neurocognitive trajectory and proteomic signature of inherited risk for Alzheimer’s disease
Source: PLoS Genet. 2022 Sep 1;18(9):e1010294. doi: 10.1371/journal.pgen.1010294 (PMC9436054; doi:10.1371/journal.pgen.1010294)
Supplement: S3 Fig — The Alzheimer’s disease polygenic score was independently validated in the Mass General Brigham Biobank. Age was assigned based on age at diagnosis of Alzheimer’s disease for those affected or date of last follow-up for others. Similar to the UK Biobank, we observe a significant gradient in Alzheimer’s disease prevalence across polygenic score deciles at later ages in a logistic regression model adjusted for sex and the first four genetic principal components. Error bars represent 95% confidence intervals. (DOCX) [file pgen.1010294.s003.docx]

**FIGURE S3: Age-stratified relationship between polygenic score and Alzheimer’s disease diagnosis in the Mass General Brigham Biobank.**


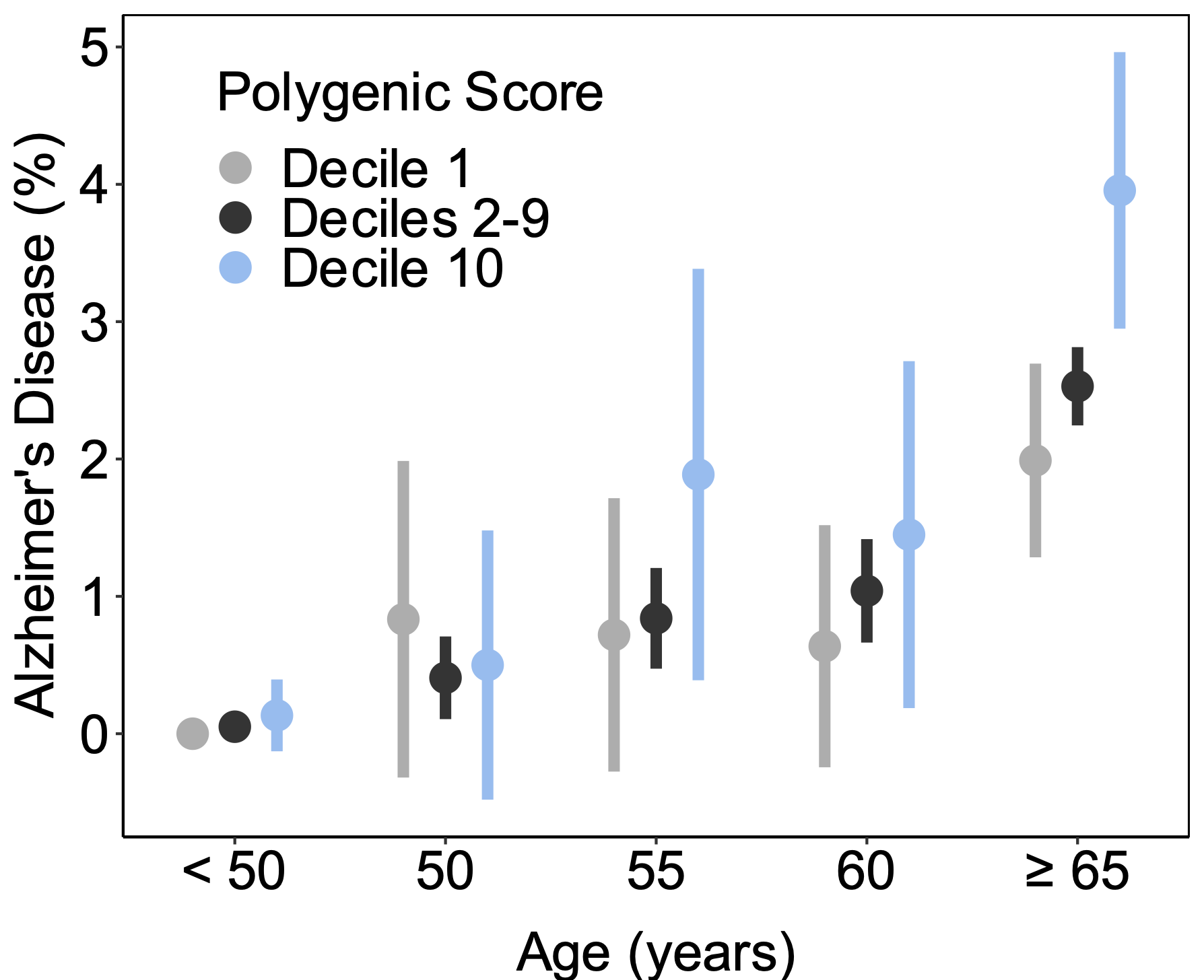


The Alzheimer’s disease polygenic score was independently validated in the Mass General Brigham Biobank. Age was assigned based on age at diagnosis of Alzheimer’s disease for those affected or date of last follow-up for others. Similar to the UK Biobank, we observe a significant gradient in Alzheimer’s disease prevalence across polygenic score deciles at later ages in a logistic regression model adjusted for sex and the first four genetic principal components. Error bars represent 95% confidence intervals.
