# Supplementary material for: Neurocognitive trajectory and proteomic signature of inherited risk for Alzheimer’s disease
Source: PLoS Genet. 2022 Sep 1;18(9):e1010294. doi: 10.1371/journal.pgen.1010294 (PMC9436054; doi:10.1371/journal.pgen.1010294)
Supplement: S4 Fig — To assess differences in protein levels among individuals <45 years (mean 32.6 years), when the onset of Alzheimer’s disease is even more unlikely, we analyzed standardized levels of the 28 proteins identified in the overall dataset. A low polygenic score indicates individuals in the first decile of the distribution and a high score indicates individuals tenth decile. * represent proteins with levels significantly different between high and low polygenic score individuals. In middle age, protein levels are consistently associated with polygenic score (p<0.05, two-tailed t-test). Whiskers represent 1.5*IQR. (DOCX) [file pgen.1010294.s004.docx]

**FIGURE S4:** **Sensitivity analysis of circulating protein levels and polygenic score in individuals < 45 years**


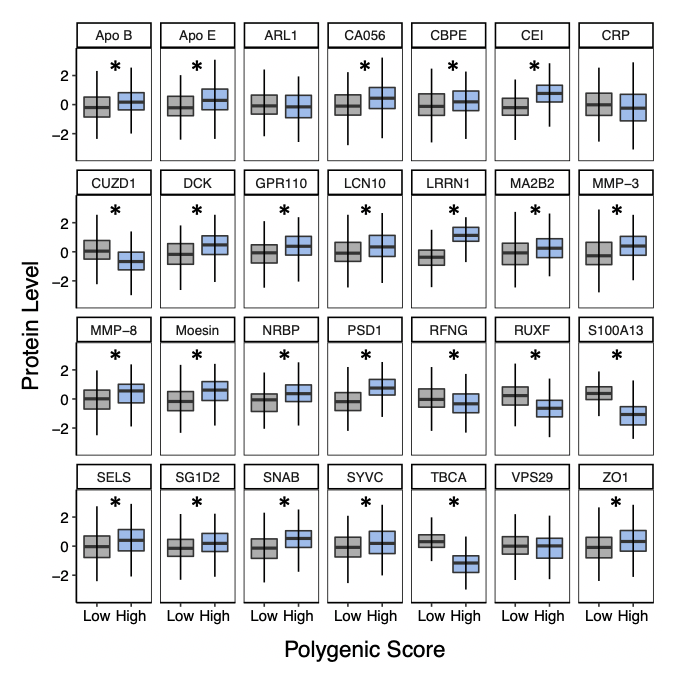


To assess differences in protein levels among individuals <45 years (mean 32.6 years), when the onset of Alzheimer’s disease is even more unlikely, we analyzed standardized levels of the 28 proteins identified in the overall dataset. A low polygenic score indicates individuals in the first decile of the distribution and a high score indicates individuals tenth decile. * represent proteins with levels significantly different between high and low polygenic score individuals.

In middle age, protein levels are consistently associated with polygenic score (p<0.05, two-tailed t-test). Whiskers represent 1.5*IQR.
