# Supplementary material for: Neurocognitive trajectory and proteomic signature of inherited risk for Alzheimer’s disease
Source: PLoS Genet. 2022 Sep 1;18(9):e1010294. doi: 10.1371/journal.pgen.1010294 (PMC9436054; doi:10.1371/journal.pgen.1010294)
Supplement: S5 Fig — Boxplots are displayed comparing levels of 8 proteins in individuals with a high polygenic score for Alzheimer’s disease (top 10%) and a low polygenic score (bottom 10%) in the MESA cohort. Of the 28 proteins associated with a high polygenic score in the INTERVAL discovery cohort, 8 proteins were available in the MESA cohort. Among the 8 proteins assayed, 7 replicated their association with a high polygenic score for Alzheimer’s disease. P values computed using a two-sample one-tailed t-test using adjusted protein levels (see Methods). Whiskers represent 1.5*IQR. (DOCX) [file pgen.1010294.s005.docx]

**FIGURE S5: Replication of polygenic score protein associations in the MESA cohort**

Boxplots are displayed comparing levels of 8 proteins in individuals with a high polygenic score for Alzheimer’s disease (top 10%) and a low polygenic score (bottom 10%) in the MESA cohort. Of the 28 proteins associated with a high polygenic score in the INTERVAL discovery cohort, 8 proteins were available in the MESA cohort. Among the 8 proteins assayed, 7 replicated their association with a high polygenic score for Alzheimer’s disease. P values computed using a two-sample one-tailed t-test using adjusted protein levels (see Methods). Whiskers represent 1.5*IQR.
